# Low-Cost Electronic Tagging System for Bee Monitoring

**DOI:** 10.3390/s18072124

**Published:** 2018-07-02

**Authors:** Paulo de Souza, Peter Marendy, Karien Barbosa, Setia Budi, Pascal Hirsch, Nasiha Nikolic, Tom Gunthorpe, Gustavo Pessin, Andrew Davie

**Affiliations:** 1 Data61|CSIRO, Sandy Bay, TAS 7005, Australia; peter.marendy@csiro.au (P.M.); boo.davie@csiro.au (A.D.); 2 School of Technology, Environments and Design (TED), University of Tasmania, Sandy Bay, TAS 7005, Australia; setia.budi@utas.edu.au (S.B.); pascal.hirsch@utas.edu.au (P.H.); 3 Vale Institute of Technology, Belém, Pará CEP 66055-090, Brazil; kr-barbosa@yahoo.com.br; 4 Faculty of Information Technology, Maranatha Christian University, Bandung 40164, Indonesia; 5 Data61|CSIRO, Epping, NSW 1710, Australia; nasiha.nikolic@csiro.au; 6 RFIT Pty Ltd., Kangiara, NSW 2582, Australia; tom@rfit.com.au; 7 Polytechnic School, Universidade do Vale do Rio dos Sinos, São Leopoldo, RS CEP 93.022-750, Brazil; pessin@gmail.com

**Keywords:** RFID, social insects, *Apidae*, *Meliponini*, insect behaviour

## Abstract

This paper introduces both a hardware and a software system designed to allow low-cost electronic monitoring of social insects using RFID tags. Data formats for individual insect identification and their associated experiment are proposed to facilitate data sharing from experiments conducted with this system. The antennas’ configuration and their duty cycle ensure a high degree of detection rates. Other advantages and limitations of this system are discussed in detail in the paper.

## 1. Introduction

Marking social insects is an effective way to identify some individuals in the colony and monitor their behaviour as they return to their nest after foraging activities. Common methods for marking insects include the use of ink (where colour and patterns are limited), wire bands, micro-dots, wing trimming, among other strategies [1]. The entomologist will record when the marked insects arrive at and leave the nest, and later correlate their observations with other parameters. More recently, image-processing-based systems have been used in making this a less labour-intensive process [2]. Limitations in physical marking persist, like the finite variations of patterns and colours that can be used, affecting the number of insects that can be uniquely marked.

More recently, electronic tagging based on RFID (radio frequency identification) technology has been used for social insects (e.g., [3,4,5]). RFID tags have proven to be valuable as they can be small enough to be glued onto insects, and unique encoding in each tag can support identification of thousands of animals in a single experiment. While RFID tags have unique identifiers from the factory, this could restrict capturing experimental details in each deployment. For this reason a specific data format has been developed and adopted (see Section 2.3.2 for details). Some disadvantages of RFID tags include:

- Cost of the tags and the supporting electronics;
- Extra weight of the tags may disturb bees, limiting their capacity to carry pollen, nectar, and water. This might also change the insect behaviour;
- Tags being lost in the process of fitting bees with an RFID tag;
- Power usage requirements, which in some cases limits the application in remote areas;
- Disturbance to the hive, including to bees without tags. As a consequence of the short communication range of the RFID tags, bees must be forced to approach electronic antennas through restrictive channels installed at the hive entrance. This certainly disturbs the hive behaviour on warmer days; and
- Not detecting bees with RFID tags as they approach a check point (hive or feeding station) equipped with the antennas. Not detecting RFID tags will compromise the quality of the experiment being conducted.

Success rate in detection are reported to vary considerably (e.g., [3,6,7,8,9], and references therein). A recent approach proposes methods to address misreadings of RFID detections using data from the entire cohort of bees fitted with those devices to infer individual behaviour [10].

In this paper, we present a RFID platform that addresses some of the key challenges of using an RFID-based system for social insect monitoring, such as misreadings, by a rigorous modelling of multiple patch antennas positioning and empirical testing of the best model arrangements. The position of the antennas is also combined with the optimal duty cycle of the reader, providing some redundancy in detection of tags.

In addition, we propose an elegant method for data storage to allow data sharing among scientists using this system, as well as a framework for Quality Assurance and Quality Control (QA/QC) to ensure data acquired is relevant and accurate.

Typical results obtained in field experiments are presented. Some known limitations of RFID-based systems are also discussed.

## 2. System Architecture

We have developed both a hardware and a software system that allows the electronic detection of bees in their natural environment, or in the lab, producing useful data about their behaviour. The architecture of the system is described in terms of its hardware components, embedded software, proposed data formats and data storage schema, and an overarching quality assurance and quality control framework. The following sections provide detail on all components of the system architecture, their importance and how they work together.

### 2.1. Hardware

The hardware components are the electronic tags, reader units, antennas, printed circuit board, and housing.

#### 2.1.1. Electronic Tags

The RFID tags used in these experiments are 2.5 mm × 2.5 mm × 0.4 mm in size, weigh 5.4 mg and are manufactured by Hitachi Chemicals [11]. The operating temperature of these tags is between −20 °C and 70 °C, and the storage temperature is between −30 °C and 75 °C. Both of these temperature ranges are valid between 10% and 80% relative humidity. We operated these tags under higher humidity conditions in the Amazon without noticeable decrease in performance. The tags respond optimally at frequencies between 860 MHz and 920 MHz.

The tags have three memory banks: a 96-bit tag identifier (TID), a 128-bit Electronic Product Code (EPC), and a 64-bit password. Each tag is registered with a unique hexadecimal identifier written into the tag’s EPC. Metadata about the bees is encoded into the EPC, capturing information about the location where the bee was tagged, what kind of platform the bee was tagged at, and species and type information about the bee. The metadata is described in more detail in Section 2.3.2.

#### 2.1.2. Printed Circuit Board

**Figure 1:**
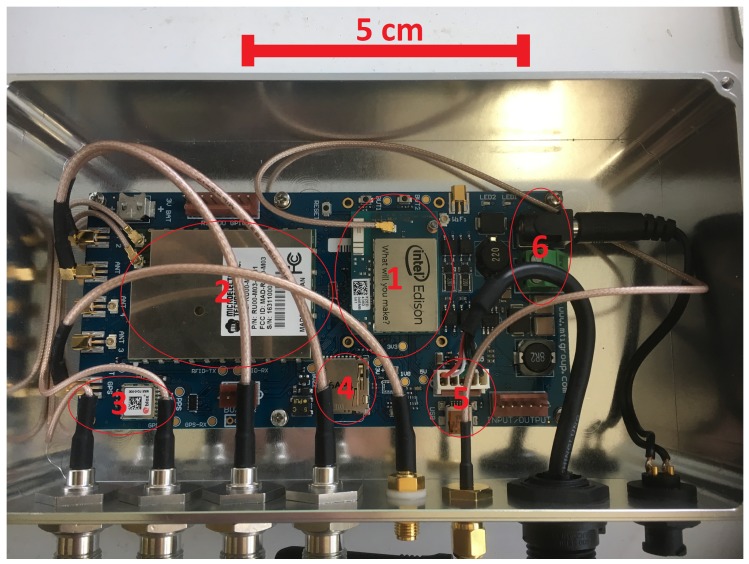
Printed Circuit Board showing the main components: (1) IoT module; (2) HP-SiP module; (3) GNSS receiver; (4) micro-SD card; (5) micro-USB I/O; and (6) power regulator.

The printed circuit board (PCB) integrates the following components into a single platform: HP-SiP module (HI-Power System in Package) (RFID reader), IoT module (Intel^®^ Edison, Santa Clara, CA, USA), GNSS receiver (Global Navigation Satellite System), micro-SD card, micro-USB I/O, and power regulator. Figure 1 shows the PCB and its major components.

The HP-SiP module supports up to 4 antennas that are multiplexed through dwell settings (see Section 3.3.2 for further details), with power levels up to 31.5 dBm, and is capable of reading hundreds of tags per second. The reader’s command set provides many commands for controlling the ISO 18000-6C (https://www.iso.org/standard/46149.html) compatible HP-SiP module. The operator can configure the module for various operations such as:

- **Inventory:** Allows the module to gather the EPC data for any tags of interest.
- **Read:** A lower-level operation that allows any specified memory bank on tags of interest to be read.
- **Write:** An operation that allows the module to write a single 16-bit word to a specified memory bank on tags of interest.
- **Kill:** An operation that allows the module to render tags of interest inoperable.
- **Lock:** Allows the permissions for the memory banks on tags of interest to be read or adjusted. The permissions are for each memory bank and include read only access, password protection, ability to adjust the settings after they have been set, and the visibility of the passwords.

The module can also be queried to determine its current state and the last error encountered.

Intel^®^ Edison is a small computer system previously manufactured by Intel for Internet of Things (IoT) development. This computer system is powered by a 500 MHz Intel^®^ Atom dual-core processor, 1 GiB DDR3 RAM, 4 GiB eMMC Flash memory, and it runs Poky Linux (http://git.yoctoproject.org/cgit/cgit.cgi/poky/about/) Linux as its operating system. It supports Python, C/C++, NodeJS, Java, and other programming languages. For network connectivity support, the Intel^®^ Edison is bundled with Bluetooth 4.0 and Wi-Fi.

The compact GNSS receiver unit from u-blox (Max 7Q module) integrates GPS, GLONASS, GZSS and SBAS (which are different global network satellite systems). The GNSS function provides location of the platform, updates local time, and confirms the HP-SiP module is operating within the ISM (industrial-scientific-medical) band, which does not require a licence, for the region where it is installed. According to the International Telecommunication Union [12], the decision on frequencies used for the ISM band is a national matter. The HP-SiP module has to be set up for each allowed frequency in the country in which it operates, and the GNSS can help to confirm the unit is operating in the allowed band. This prevents the risk of a kit configured for one country being operated using prohibited RF bands in another country.

The micro-USB I/O provides access to the IoT module. It is an efficient gateway to communicate via wired serial connection with a field laptop. It also provides safe access to stored data in case the wireless communication fails. The micro-SD card can be used to store large amounts of data and effectively work as a backup. If other communication gateways fail (Bluetooth, Wi-Fi or via micro-USB) physical access to the card will ensure data from the experiments can be recovered.

The power regulator enables the system to work from a variety of DC power sources ranging from 6 up to 60 V without any additional configuration.

#### 2.1.3. RFID Antennas

**Figure 2:**
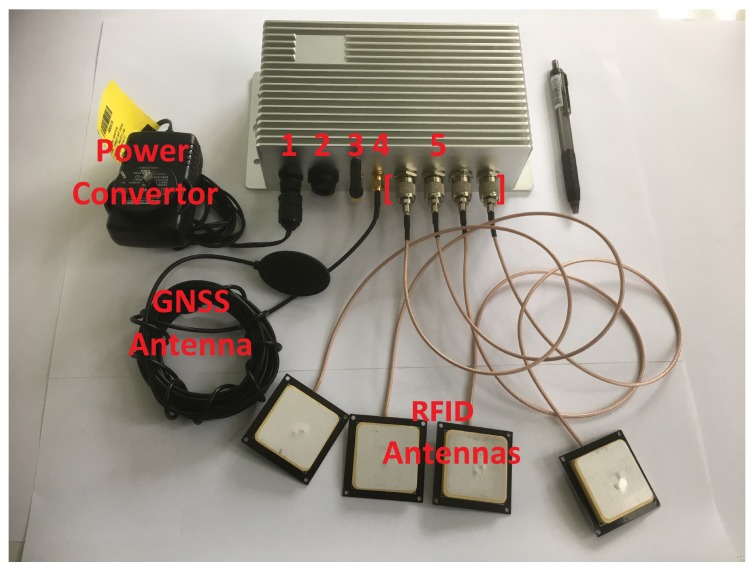
Aluminum housing (18.5 cm × 10.5 cm × 5.5 cm) and peripherals. (1) 4-pin power inlet; (2) micro-USB port; (3) Wi-Fi antenna; (4) GNSS antenna, and (5) RFID antennas and ports.

The antennas, as seen in Figure 2, are RFIT 2DBI-1: 2 dBi ceramic patch antennas with TNC male connectors. The ceramic patch is 40 mm × 40 mm × 5 mm, centered on a 50 mm × 50 mm × 1 mm ground plane. These antennas have been used with 50 cm, 75 cm and 100 cm cables. The system can also work with other antennas such as the Taoglas 49.5 mm × 49.5 mm × 7.5 mm ISPC.86A 868 MHz Ceramic Patch Antenna, 92 mm RG-178 (http://www.taoglas.com/product/ispc-86a-868mhz-ceramic-patch-antenna-49-5mm/) which has been used in Europe.

#### 2.1.4. Housing

The two-part housing for the PCB is manufactured from aluminum. It includes a built-in heat sink as part of the enclosure to provide cooling for the PCB and electronics. It also provides several external connectors including:

- Four TNC antenna connectors;
- An SMA antenna connector for GNSS;
- An SMA antenna connector for Wi-Fi;
- A Micro-USB Type B connector;
- A 4-pin power connector.

#### 2.1.5. Power Requirements

The system can operate with a solar panel of 120 W, with a 12 V 33 Ah battery. A 12 V regulator and a 12 V to 5 V converter are used for powering other lower-voltage instruments.

The specification provided by the manufacturer (MTI) of the HP-SiP module indicates a peak power draw of 15 V–20 W when the RFID is running at maximum power (transmitting at 2 W RF). We have observed (0.2 V–0.9 A at 12 V when the inventory cycle is running) a need for no more than 10.8 W, amounting to an absolute maximum of 10.8 W × 24 h = 259.2 Wh per day. This energy can be provided by commercially available solar panels, which allows the system to operate in remote areas.

A fully charged 12 V 33 Ah battery is sufficient to run the system, with full inventory cycle running continuously, for one and a half days if the solar panel is disconnected. We have run this configuration, with solar panel and battery, uninterruptedly in Tasmania through winter with approximately four and a half full daylight hours equivalent. This includes cloudy days for a period longer than a week.

### 2.2. Software

In this section we describe three major components of the software system: the embedded software, communication gateways and watchdog system.

#### 2.2.1. Embedded Software

This component is the software that controls and communicates with:

- HP-SiP module:
  - issuing commands (e.g., initialization of the module including the region specification, duty cycle (described in greater detail in Section 3.3.3))
  - antenna configuration and receiving responses (e.g., detections of RFID tags, data on temperature of the module, signal strength indicators of each antenna).
- GNSS unit: initializes the unit, reads responses and makes use of the data as needed. Experiments with movable feeding stations or hives could also be supported with this component.
- Memory storage devices:
  - writing data into memory and retrieving when required, such as by the QA/QC framework
  - Data management including compression and transmission.
- Power regulator: checking battery levels.
- Other hardware components: verifying the operational status of the hardware including communication gateways and registered its status in log files and provides input to the QA/QC framework.

The embedded software, written in Python 2.7, utilizes multiprocessing as the Edison^®^ IoT module has a dual-core processor. The embedded software generates daily log files containing detailed information about the operation of each unit of the hardware and other relevant information. This software can be further customized to accommodate specific user needs.

#### 2.2.2. Communication Channels

The availability of Wi-Fi allows the system to be accessible remotely via SSH (secure shell) or SCP (secure copy) and the data to be retried as needed by users. For example, a user might want to receive experimental data every day via e-mail or if a specific bee is detected. During field inspections the user can access data via Bluetooth and this communication channel can also supports visualization tools such as augmented reality (e.g., [13,14].) To operate communication channels the user can use the embedded software, Linux commands or external devices.

#### 2.2.3. Watchdog

To ensure the continued operation of the embedded software, a system watchdog is enabled. The watchdog listens for a notification from the embedded software arriving within a specified interval. If the notification is not received within the interval, the watchdog restarts the system service for the embedded software. The watchdog provides assurance that, should the embedded software fail, it will be restarted promptly. This re-initializes the hardware as part of the start-up process for the embedded software and recommences regular operation. The watchdog is configured for a 30 s interval.

### 2.3. Data Format

Tagged bees passing by antennas at hives or feeding stations are registered by the reading platform. Consider a bee detected leaving its hive, and a few minutes later arriving at a feeding station; there are separate registered events for the detection of this bee at each location. A file is created daily for each platform, containing the time each bee visits that platform. The following sections describe the data formats used for the reading platforms and for the RFID tags, as well as the strategy for the data storage and the database schema. This section describes the data format for both reading platforms and RFID tags.

#### 2.3.1. Data Format for Reading Platforms

Each platform creates a CSV (comma-separated values) file every day, containing the times at which each bee was detected. The name of the file has the following format: YYYYMMDD_CCSSSTTNNN with extension .csv, as described in Table 1.

Some examples of file names are given in Table 2:

#### 2.3.2. Data Format for RFID Tags

Each RFID tag has a unique code that, when detected at a given time, is recorded in a file named as described in Section 2.3.1. Each RFID tag is programmed, prior to being fitted on a bee, with a unique 24-digit hexadecimal code in the format CCSSSTTNNNXXXYYZ****BBBB as described in Table 3. There is one exception; a special-case testing tag that uses the code FFFFFFFFFFFFFFFFFFFFFFFF. This tag is used manually to verify that the system is operational and to create a persistent registry of user visits to the units. Details are provided in Section 3.2.

Some examples of RFID tag codes are given in Table 4:

### 2.4. Data Storage

Reader recordings are stored one per line. Each line registers the detection time in UTC (coordinated universal time) in seconds (or at higher resolution if required), the bee visiting that platform (as described in Section 2.3.2), and the detecting antenna. Table 5 presents a sample of CSV file content.

These data, providing information on the activity of bees leaving and returning to the hives, and visiting an artificial feeder, were processed in time-order and analyzed to reconstruct foraging behaviour [4].

The system offers internal storage for the files and logs that are produced. The micro-SD card provides extended external storage that can be used in place of the internal storage. A timed script runs daily to compress the data files and the log files that are more than two days old. This reduces the likelihood of the system running out of storage space, although it has occurred when the user has not cleared the memory. For that reason memory status has been included in the QA/QC framework see Section 2.5.

An alternative approach to reduction of storage requirements is the use of a SQLite version 3 database, implemented within the embedded software, to consolidate and filter tag data before writing to the SD Card. After tag records are written to the SQLite database, a consolidated and filtered version of the data is written to the SD card.

The combination of two internal locations to store files, an SD Card, and access to external data-stores through online services (when Wi-Fi is available to the system) offers flexibility in data storage. The current status of memory resources is reported through the QA/QC process as described at Section 2.5.

The data can be accessed via Wi-Fi, Bluetooth, and direct cable connection. When the system is connected to Wi-Fi with a known IP address, the data can be recovered using a secure protocol such as Secured Copy (SCP). This technique has been used successfully, with an automated script running on a daily schedule fetching any new files found on the system. A SCP client on a laptop, and a cable connection to the system, also allows the data to be recovered directly by an operator. The system also offers a smaller storage area that is exposed as a USB drive when a cable is connected to the system. This can be used as an alternative way to recover the data using a cable connection.

The database schema has been designed with components, samples, and annotations as the central elements. Examples of components are weather stations, hive scales, in-hive sensors such as humidity and temperature sensors, and our system. Data recorded by a component is, for the database schema, a sample. Annotations are notes made by users that are associated to the experiment, the components, or the samples. Examples of annotations are the consumption of fluids in a feeder, the presence of other insects around the hives, weather events, calibration of sensors and simple comments on the data.

This schema provides a flexible and extensible design that allows additional features to be added while facilitating complex searches on the data. It has also been used in the design of a collaborative framework for immersive analytics [14]. This schema allows for assets, experiments, and data to be recorded, annotated, and searched.

A PostgreSQL 9.4 (https://www.postgresql.org/) implementation of the schema is used to store the experimental data. The live implementation includes tables to capture any changes, and their source (who/what), made to the core tables in the schema via SQL Triggers. This allows users to review changes and roll back to previous versions where required or desirable. The ability to roll back is particularly applicable to annotations and observation descriptions.

A data storage strategy implemented in the system is important because it allows data to be recorded, compressed, transmitted and stored effectively. As a result the device will not lose data or run out of memory, and the data management will be efficient.

#### Data Curation

Data curation is an important part of the experimental process. Human mistakes in the conduct of experiments can occur. This includes, for example, tags designated for one hive being fitted to bees from another hive. Such errors can be noted and corrected during the curation process. It is important to process any data to ensure that the correct values are being stored. The raw data is retrieved from the system and written to a data-store. These files are kept as the ground truth. Once collected, the data follows a three step curation process:

- **Data Verification** confirming the operator has used the correct tags in the correct hive, checking expected tag times, assessing any potential misreadings, verifying if any bee with a tag is dead near an antenna causing spurious detections.
- **Injection into a Database** scripts write the processed data into a database to facilitate ease of search.
- **Data Archival** data is made available as a collection of verified CSV files via a data repository.

### 2.5. Quality Assurance and Quality Control (QA/QC)

A quality assurance and quality control system runs autonomously to ensure integrity of the experiment and flag potential issues with the system. Its architecture is divided into two major components that assess the quality of engineering data and of the scientific data [15]. As an outcome it reports the working status of the system and if the quality of the experiment could be compromised at the current moment or in a near future.

#### 2.5.1. Engineering Data

Engineering data includes information about the working status of some components like:

- **GNSS:** Verifies if the GNSS is operational and ensures there is no clock discrepancy between the microcomputer and the GNSS. Also verifies if the RFID reader is operating within the allowed local ISM-band.
- **RFID reader unit:** The unit has several low-level error reports that can be interpreted and addressed at a higher level.
- **Wi-Fi:** Confirms if this gateway is operating as expected.
- **Memory:** Assesses the amount of memory used, that is still available, and when it is likely to become an issue estimated from the reading frequencies.

The key advantages of having QA/QC for engineering data is to assure the user the system is operational and any issues are detected and promptly reported.

#### 2.5.2. Scientific Data

The QA/QC of scientific data allows the operator to verify that their experiment is recording data as expected, and produces warnings about anomalous data and behaviour. This component automatically analyzes the data produced by the system, including:

- **Anomalous detection of bees:** An example is a bee that is detected leaving the hive twice without returning. It could occur when the bee finds another entry to the hive (which should be immediately fixed) or if the RFID readers stop working for some time. Another potential cause is a less than ideal duty cycle, which could allow bees to move on the antennas without being detected (see  Section 3.4.3).
- **Bee mingling:** bees visiting neighbour hives is an expected behaviour, but ideally this should be highlighted.
- **Detection within antennas:** for the honey bee configuration, two pairs of antennas are recommended (see Figure 7a). The number of detections of tags is highly dependent upon bee behaviour, and hives equipped with the system should present similar results as they are subjected to similar environmental conditions. It is acceptable for bees to be detected more frequently in one antenna pair than in the other, but all hives should show similar results.

The QA/QC framework for engineering or scientific data can be updated and expanded depending on specific needs of users. The main advantage of having a QA/QC framework is that issues can be promptly identified, prioritized based on the consequences and addressed.

## 3. Operating Methods

This section describes how to operate the system, including how to fit bees with RFID tags, how to set up and test the system, its antenna configuration, and expected issues.

### 3.1. Tagging Bees

**Figure 3:**
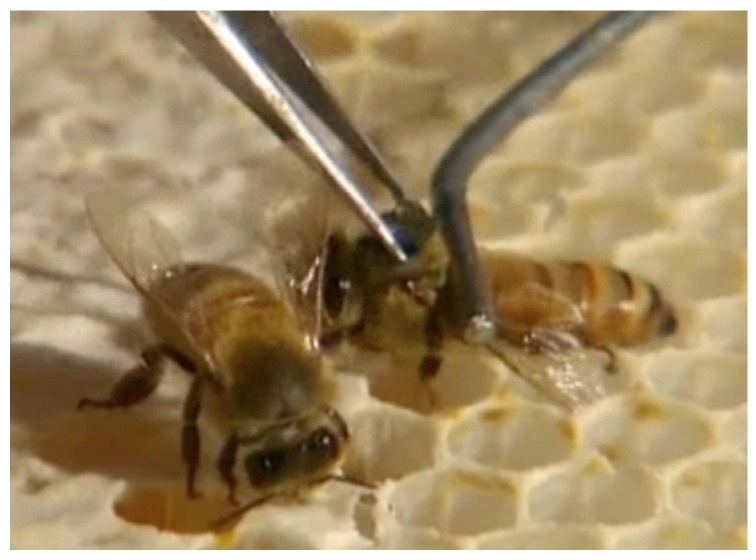
RFID tag being fitted to the thorax of a honey bee. This process can be done in the hive with non-aggressive bees.

RFID chips are dipped in one-component cyanoacrylate (Cyberbond^®^ 2610) and, while holding the bee lightly with a pair of forceps at the upper abdomen (holding down the wings), the RFID chip is placed between the wings at the top of the thorax. The bee is released after a few seconds (Figure 3). More aggressive bee strains like the Africanized honey bee make it nearly impossible to work around open hives. In that case bees are collected and held manually during the RFID fitting or several bees are brought to the laboratory where CO${}_{2}$ or cooling can be used to decrease bee movement. Depending on the nature of the study, such as checking longevity of bees, it will be ideal to tag bees as they hatch in their first day of life as an adult. Frames of late instar capped brood can be placed in incubators kept at an appropriate temperature (about 35 °C for honey bees) and the bees can be tagged as they emerge. Newly emerged bees are not aggressive nor can they fly for the first day or two. Forager bees can be tagged directly on feeding stations, but in that case the bee could be from a wild hive.

**Figure 4:**
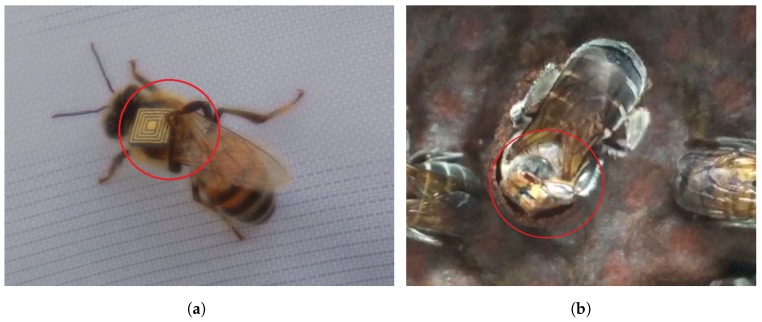
Example of bees disturbed by the RFID tags. (**a**) *Apis mellifera* trying to remove the RFID tag; (**b**) *Melipona melanoventer* trying to remove the RFID tag.

RFID tags can be lost in the process of tagging bees. We observed retention rates varying from as little as 20% to as much as 90%. Low retention rates can be caused by poor quality glues as well as high moisture and temperature where the hives are located affecting curation times, how experienced those attaching tags on bees are and how aggressive the bees are. Bees can also remove the tags (Figure 4).

### 3.2. Testing the System

An efficient way to verify if the system is reading RFID tags and processing the data accordingly is to use one RFID with a known identity code different from those used for bees. For this purpose all experiments are using a tag with a hexadecimal sequence of 24 *F*s (FFFFFFFFFFFFFFFFFFFFFFFF) in the EPC memory. This tag is mounted on a non-metallic rod which is inserted manually at the hive entry, triggering a messaging task. Alternatives tested are:

- **micro-USB connection:** Using a laptop the operator can confirm the system is working by using the testing tag and verifying if the file was updated. In this case, the Wi-Fi and the Bluetooth connection will not be tested.
- **Bluetooth:** an augmented reality system, MelissAR [13], has been developed for visual analytics of honey bee behaviour in the field. The implementation was realized on a Sony Xperia Z4 which utilizes Bluetooth to communicate with the system to recover hive data.
- **e-mail:** an electronic message can be sent from the system via Wi-Fi either directly to recipients, as an email, or to a RESTful service, which then generates email messages to subscribers, to confirm the system is operational.
- **SMS:** an electronic message is sent from the system via Wi-Fi to a RESTful service which then generates SMS messages to subscribers.

### 3.3. System Set Up

The system set up will depend on the bee species under study. In this paper we will describe the set up for the honey bee (*Apidae*). Their hive entrances are wider than for other bee species like *Bumbus*, *Xylopcopa*, *Euglossini* or *Meliponini*.

#### 3.3.1. Antenna Physical Arrangement

**Figure 5:**
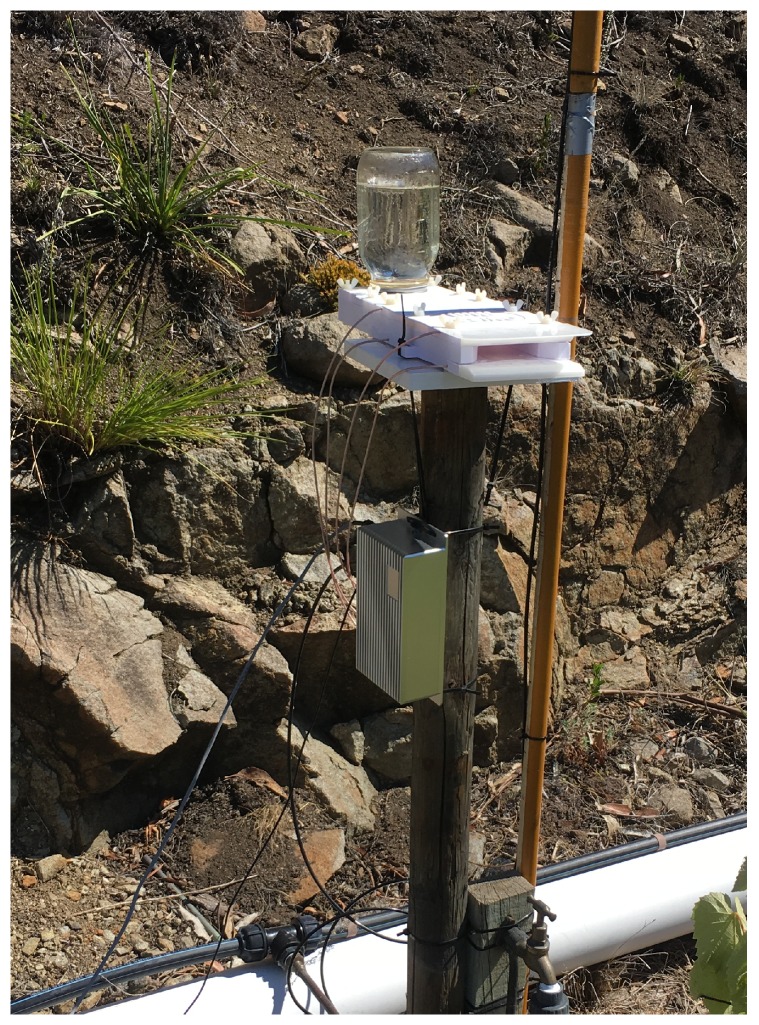
Feeder station equipped with the presented system.

The direction of bee movement (in or out of their nest or feeding stations) can only be determined by using at least two antennas. The use of only two antennas must be associated with some constraints in bee path to ensure a minimum distance between the antenna and the tag attached to the bee allowing its detection. Using two pairs of antennas has proven to be more efficient as a result of cross antenna excitation. Details of this feature are described in Section 3.3.3. Figure 5 shows a feeder station equipped with the presented system.

**Figure 6:**
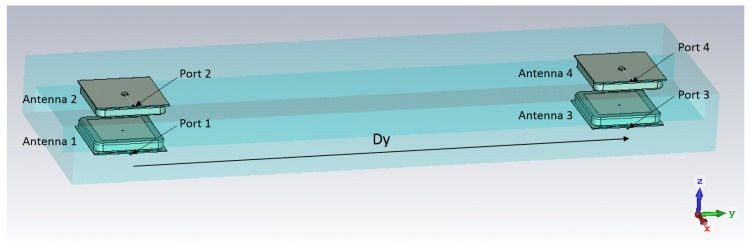
Four patch antennas placed on the entrance housing. Patch antennas are fed by a coaxial probe. The antenna size is 50 mm × 50 mm × 6.5 mm.

**Figure 7:**
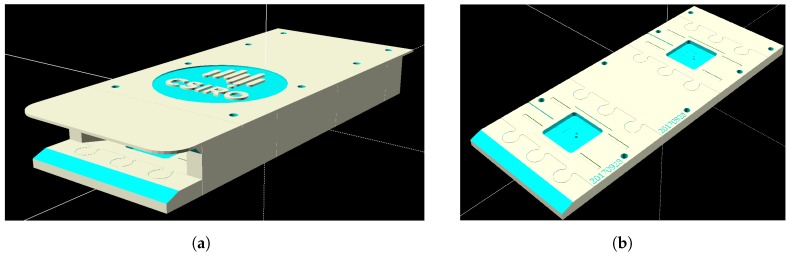
Model of a 3D-printed housing for the RFID patch antennas. This particular model has proven to be useful for *Apis mellifera*. Other bee species may require different arrangements. (**a**) External view of entrance housing. The structure is 38.5 cm × 12.5 cm × 4 cm; (**b**) Internal view of entrance housing. This is mirrored by the opposite layer of the housing to provide matching pairs of antennas. The centers of the highlighted squares (where the antennas are mounted) are 17 cm apart.

The patch antenna with coaxial feed was designed with CST Microwave Studio (CST Microwave Studio 2018 User Manual [Online]. Available: www.cst.com.). The antenna parameters are optimized to achieve a resonant frequency at 875 MHz. A substrate with high permitivity is selected to reduce the size of the antenna and the substrate thickness is increased to increase the antenna bandwidth. Several configurations were investigated both experimentally and by CST modelling and include: shifting antennas 1 and 3 in +x-direction and antennas 2 and 4 in −x-direction, shifting antennas 1 and 4 in +x-direction and antennas 2 and 3 in −x-direction etc. Both experimental and simulated results show that the best coverage has been achieved when all antennas are placed along the symmetry plane of the box, as shown in Figure 6. The spacing between the pairs of patch antennas in y-direction was varied to simulate mutual coupling between pairs of patch antennas placed on the antenna housing (Figure 6 and Figure 7a,b). As expected, the coupling between Port 1 and Port 3 has a maximum value close to the resonant frequency of the patch antennas and it reduces by increasing the distance Dy. The spacing Dy = 170 mm has been experimentally selected as a compromise between the spacing required for achieving sufficiently low mutual coupling between antenna 1 and antenna 3 and the practical size of the box.

**Figure 8:**
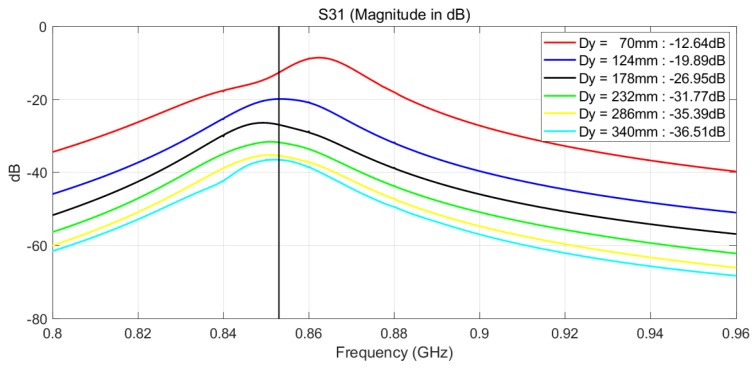
Simulated S31 versus distance Dy for the configuration of the antennas shown in Figure 6. Distance Dy is measured between the centres of patch antennas.

The simulated coupling between Port 1 and Port 3 versus distance Dy is shown in Figure 8. The spacing between the antennas was increased from 70 mm to 340 mm, or 0.20 $\lambda_{0}$ to 0.97 $\lambda_{0}$ calculated at 875 MHz. As expected, the coupling between Port 1 and Port 3 has maximum value close to resonance frequency of the patch antennas and it reduces by increasing distance Dy. The coupling between Port 1 and Port 4 shows similar trend and it is on average by 3 dB lower than the coupling between Ports 1 and 3. The coupling between Port 1 and Port 2 is strong since the corresponding patch antennas are facing each other and it is close to −8.5 dB.

In this experiment, the antennas are spaced by 170 mm in order to reduce the coupling between them. In future, other options for reducing mutual coupling while keeping the spacing between will be considered. For example, the mutual coupling may be reduced below 45 dB for centre to centre spacing of 0.33 $\lambda_{0}$, by introducing simple resonant slots on the common ground plane between two microstrip antennas [16,17].

#### 3.3.2. Antenna Electronic Configuration

The MTI HP-SiP module supports up to 16 logical antennas mapped to 4 physical transmit/receive ports. The HP-SiP module may retrieve status and configure several parameters on a per-logical-antenna-port basis. This allows for flexible configuration of the antennas through enabled/disabled states, power levels, dwell times, number of inventory cycles, and logical to physical antenna port mapping.

The module allows the antenna power level to be set in 0.1 dBm increments in the range 0 to 33 dBm. This range represents the programmatic specification for the HP-SiP module, however the highest level is not guaranteed and it allows for a ‘calibration factor’ so that a guaranteed and equal level is presented on each port. Allowing for up to 1.5 dBm in cable and connector loss gives the module an effective limit of 31.5 dBm on each antenna port. The experimental configuration uses 27 dBm to help reduce antenna cross-talk and as a result improve the quality of detections. The antennas used are a 2 dBi ceramic patches, meaning the antenna has a RF gain of 2 dBm. When the power has been set to 27 dBm, the ERP (measured at a specific point) is 29 dBm.

The combination of the configuration for dwell time, which specifies the number of milliseconds that tag protocol operations (as described in Section 2.1.2) may spend before switching to the next enabled antenna port, and inventory cycles, determines the conditions under which the port switch will occur. The dwell time can be set between 0 ms and 65,535 ms. Likewise, the number of inventory cycles can be set between 0 and 65,535 cycles. In all combinations of dwell time and number of inventory cycles, the logical antenna will remain active until the maximum number of tags have had the protocol applied, or the operation is explicitly cancelled. If the dwell time is set to 0 ms the logical antenna must be configured with at least 1 inventory cycle. Under this configuration the logical antenna will remain active until one of the general conditions is met or until the specified number of inventory cycles has completed. If the dwell time is not 0 ms but the number of inventory cycles is, then the logical antenna remains active until one of the general conditions is met or the dwell time expires. If both the dwell time is above 0 ms and the number of cycles is 1 or more, any of the previously mentioned conditions will end the logical antenna activity.

#### 3.3.3. Duty Cycle

**Figure 9:**
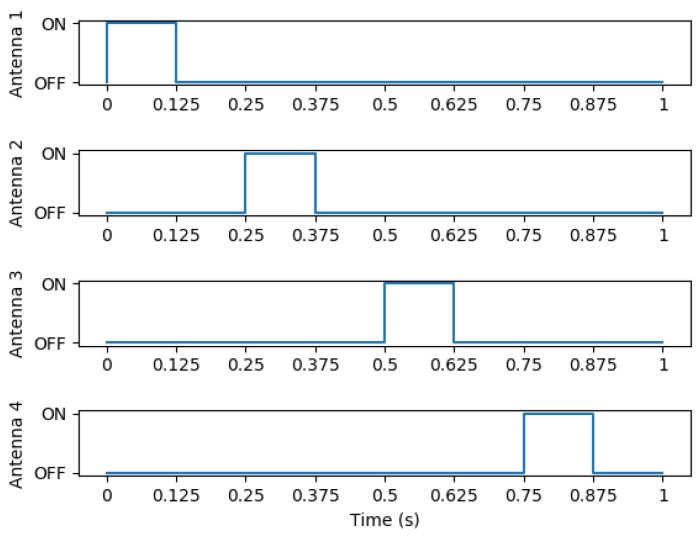
The structure of a duty cycle of the RFID reader operation. It is only possible to power one antenna at a time and all antennas must be off for a given time as well. The time span during which each pair of antennas (Figure 7) operate can be tailored to how fast the bee species under study moves.

The experimental configuration uses dwell times of 0.125 s on 8 logical antennas’ activity times mapped to 4 physical antenna ports. The logical antennas are alternately set with a 27 dBm or 0 dBm power level, i.e., logical antenna (LA) 1: 27 dBm, LA 2: 0 dBm, LA 3: 27 dBm, LA 4: 0 dBm, LA 5: 27 dBm, LA 6: 0 dBm, LA 7: 27 dBm, LA 8: 0 dBm. Logical antennas 9–15 are disabled. The resulting signals from this configuration can be seen in Figure 9.

#### 3.3.4. Example of Results

This section presents some field results obtained with the system. We present data of a single bee on a particular day, a distribution of time bees spend at the hive entry, and the activity of bees from a given colony over a long period of time. To demonstrate the system efficiency in detecting bees with some (desired) redundancy, a distribution of how many times a bee is detected in every event is also presented.

#### 3.3.5. A Day of an Individual Honey Bee

An example of a bee’s activity over the period of a day is presented in Table 6 in three level of details: raw data consisting of time the bee was detected in which antenna, as referenced by Figure 6, data filtered by events determined by having more than one minute between detections, and an interpretation of what the bee did in that event.

#### 3.3.6. Bees at the Hive Entry

**Figure 10:**
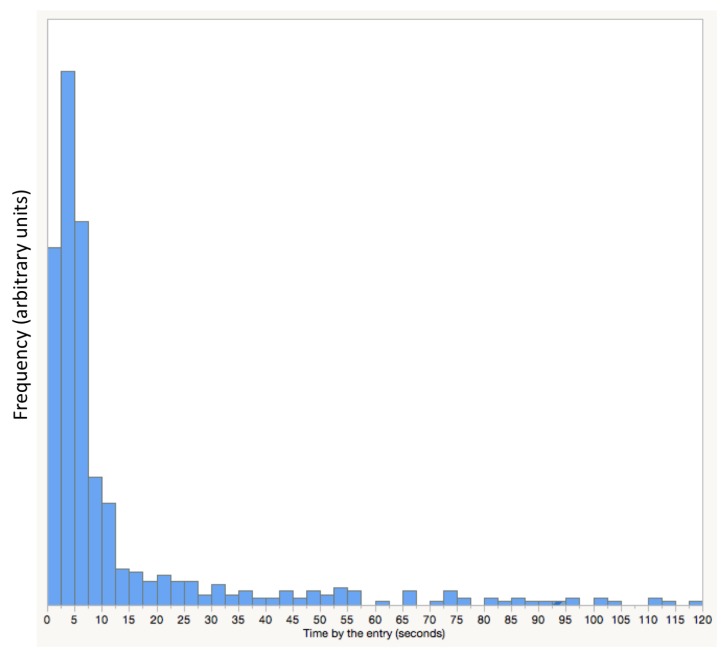
Distribution of duration worker honey bees were detected at the entry of the hive. The graphic shown has a period limited to two minutes, however some bees can stay longer.

Data can be classified by the duration of foraging or other behaviours of the bee such as staying at the entry. Figure 10 shows the distribution of time periods honey bees are detected at the entry of the hive. This data was obtained from 352 bees during 24 days. The distribution reveals that most bees will stay by the entry for no longer 15 s before entering or leaving the hive.

#### 3.3.7. Bee Colony Behaviour over a Period of Time

**Figure 11:**
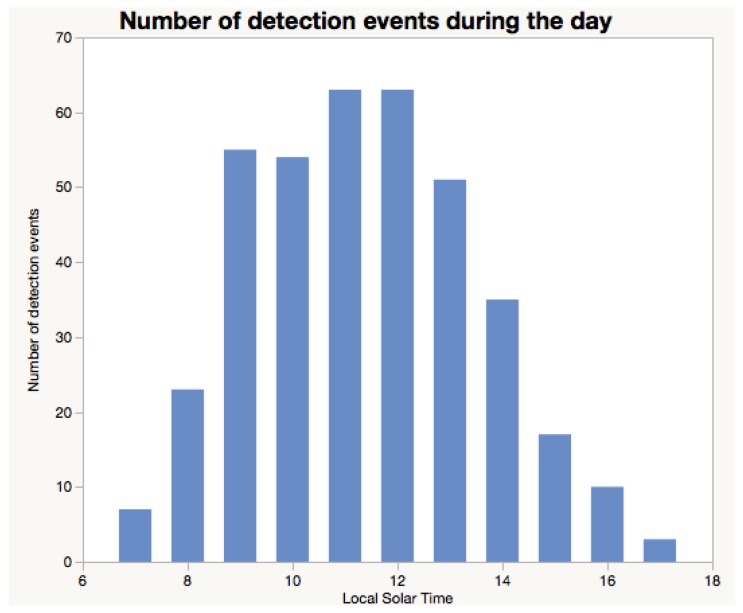
Activity of bees during the day. These data are from honey bees from 352 bees over the period of 24 days.

Figure 11 illustrates the activity of honey bees using our RFID system. This Figure combines activity data (such as the one shown in Table 6) from 352 bees during a period of 24 days.

For Figure 11 a period of one minute or more between detections was classified as an event. This means that a bee must not be detected for a minute or longer to constitute an event. This period of time is selected as part of the data interpretation process.

#### 3.3.8. Redundancy in Detection

**Figure 12:**
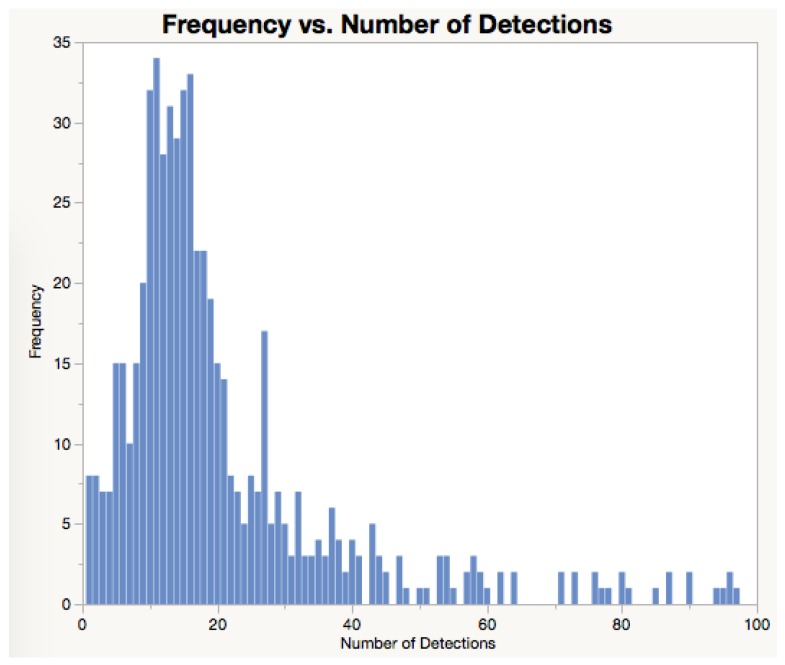
Number of detections registered per event. This data illustrates that bees are expected to be detected several times as they move near RFID antennas.

Detecting a bee more than once is desirable as it will ensure the bees are certainly registered when returning or leaving the hives, when staying at the entry of a hive, or visiting feeder stations. In the data presented in Figure 11, each event resulted in 7.14 detections on average. This number of detections per event demonstrates how well the system performs in detecting bees. This redundancy in bee detection is ensured by both the optimal positioning of the antennas and the duty cycle set for the RFID reader. Figure 12 presents the number of detections obtained per events.

### 3.4. Known Issues

While RFID is a useful technology to follow bee activity, it has some important issues the user must pay attention to including how to fit RFID tags onto bees, the consequences of dead bees with tags at the entry, reading-frequency setup and details on data interpretation. This section discusses these issues in some detail.

#### 3.4.1. Fitting RFID on Bees

During the placement of the RFID tag on a bee it is possible that a misplacement of the tag will prevent the bee from being able to fly (e.g., RFID tag touching one of the wings), a tag could impair the bee’s navigation (if it is covering their eyes) or adding the tag could hurt the bee when pressing on its thorax. Some bee species (e.g., *Melipona seminigra*) are very sensitive to the glue’s smell. Once the bee receives the RFID tag it will immediately be attacked by its own nest. In this case, it is only possible to work with these bees by placing them overnight in a separate box and releasing them the next morning. There are different strategies to be used while attaching the RFID tags to the bees. These are a result of lessons learned from entomologists and beekeepers using this and previous generations of this system, and include:

- **Bees tagged at the hive:** distress to the entire hive, no guarantee the bee will survive many days, and there is no knowledge of the role of that bee in the colony.
- **Bees tagged at the feeding station:** Bees from non-instrumented hives from the neighborhood could visit the feeder. It is very likely the bee will return to the feeder, as long as quality sugar water solution is continuously provided.
- **Bees tagged in the lab:** stress to the individuals.
- **Bees tagged as they hatch:** ideal scenario for most studies. The issue will be the uncertain future of that bee. Young honey bees usually remain in the hive for about three weeks until they start flying.

#### 3.4.2. Dead Bees at the Entry

Sometimes dead bees that have been tagged can be found near the entry of the hive. As a consequence, dead bees with RFID tags are for a period of time deposited near the reader and detected continuously. Large files will be recorded as a consequence of the presence of a dead bee with a RFID on an antenna.

#### 3.4.3. Reading Frequency

A duty cycle that is not well-designed could lead to bees passing by the antennas undetected. Ideally, the reading frequency should allow multiple detections of a passing bee. This will result in a desired redundancy which ensures the bee presence is recorded and then confirmed by a second detection. However, higher reading frequency will consume more power and memory. It is also possible to implement an adaptive reading frequency that takes into account the weather conditions and likelihood of bee activity (e.g., [18]).

#### 3.4.4. Data Interpretation

While RFIDs provide an accurate way to determine bee movements, individual bees may respond to specific hive needs such as air cooling, cleaning, and defense. These activities are usually conducted at the entry of the hive, which could lead to a large number of readings as the bees stay in the vicinity of an antenna for some time, or move intensively across the hive entry. As a consequence, data interpretation can be difficult and would require the assistance of an entomologist who has observed bees during that period of time or who is familiar with those behaviours. Post-experiment data processing could facilitate cleansing or lead to misinterpretations. While it is tempting to process the data, it is important to confirm the cleansing has produced desired results.

## 4. Discussion

The hardware can be installed in hives as well as in stand-alone feeding stations separate to hives.

Tracking bee exits and returns to hives could provide insights into whole-of-colony response to specific needs, changes in individual role as the insects mature, foraging activity of the hive, drone behaviour, and absence of the queen from the hive (e.g., nuptial flight or swarming).

Monitoring bees in feeding stations allows the observation of foraging patterns, independent of their hives of origin. Bees, visiting feeding stations usually filled with sugar water solution, can come from any hive nearby and bees from different species could also be present (e.g., *Apis melifera* and *Apis cerana* in Queensland, or *Apis mellifera* and *Bombus terrestris* in Tasmania, both in Australia). In this case, bees will be fitted with RFID tags when visiting the feeding stations and special care should be taken to avoid mixing the tags from one species with the those prepared for another species. Feeding stations also allow the exposure of bees to chemicals (e.g., pesticides) in controlled doses, and multiple feeding stations distributed over a region could contain different doses of chemicals, with control feeding stations containing none. In this case, environmental factors will be nearly identical for all bees within the region.

The number of bees to receive RFID tags will depend on the nature of the experiment. For example, to observe the behaviour of a small honey bee hive (with some 15,000 individuals), at least 200 bees should be fitted with RFID tags. Hive replication is always desired, if collective behaviour of the colony is relevant.

Additional hardware such as weather stations and hive scales can be relevant and their installation should be considered. If the user is visiting the site regularly to deploy additional RFIDs on bees or for site maintenance (e.g., grass cutting), it is always good to record observations on bee activity, presence of other insects such as wasps, ants or other bee species, pests such as varroas or hive beetles, movement of drones, and colour of pollen foraging bees are carrying. All this information can be relevant later on when the user is trying to interpret the data on individual or collective behaviour of bees with RFIDs.

The interpretation of bee activity, such as demonstrated in Table 5, should be ideally performed by an entomologist or by an experienced beekeeper. In this process of interpreting the activity of individual bees, the specialist would consider all information available about the bee species and experimental protocols adopted. That Table illustrates data obtained from a drone. Drones are expected to stay at the entry of a hive for a long period of time. It is also clear that the number of detections per event can be relatively large (see Table 5 and Figure 12). This is a desirable outcome as it confirms the system is robust to misreadings (i.e., bees passing by antennas and not being detected). Misreadings in RFID systems can be caused by several factors including distance between the RFID tag and the antenna, power shortages, and inappropriate duty cycle set up ([10], and references therein).

### 4.1. Limitations

Scientists working with RFID-based technologies are making assumptions that must be understood as they are also common to all similar electronic monitoring systems.

#### 4.1.1. Disturbing Bees

Bees could be impacted by the process of attaching RFID tags to their thorax (see Figure 4a,b). As discussed previously in this paper, these impacts are the smell from the glue affecting the acceptance of the bees in their own hives or their ability to properly forage, the stress on bees receiving the tag, glue spilling on bee’s eyes or wings, effects on bee mobility within the hive, to mention a few. Also, there are studies (e.g., [19], and references therein) suggesting the interference of electromagnetic radiation in bee behaviour.

While studies with electronic tagging of bees are designed to compare bees carrying the same tags exposed to stressors with those not exposed to those particular stressors, it is assumed that the behaviour of bees without tags exposed to and not exposed to those stressors can be translated without further analysis. This assumption will fail if bees carrying electronic tags will respond differently to the effects of stressors than bees exposed to the same stressors and not carrying tags.

#### 4.1.2. Hive Entry Modification

The hive entrance and antenna housing (as illustrated in Figure 7a) can impact the behaviour of the bees when they are trying to regulate the internal conditions of the hive such as humidity and temperature. The distance between pairs of antennas should be at least 17 cm which makes the entry relatively long. If the distance is shorter than that there will be interference across pairs of antennas. This long entry can be prohibitive to some experiments in very hot environments. The distance between a pair of antennas may be reduced by optimizing a pair of antennas which share the same substrate and ground plane.

#### 4.1.3. Vulnerability to Predators

Bees with RFID tags could be more visible to predators such as other insects and birds. Depending on bird species in the region, ethics application could be needed because of the potentially hazardous ingestion of RFID tags. If bees with RFIDs are more vulnerable this could affect the accuracy of the studies of bee longevity.

## 5. Conclusions

One of the key challenges in using RFID-based systems for social insect monitoring is the occurrence of misreadings, i.e., when the insects pass by check-points without being detected. This was addressed in our system by a combination of multiple antennas, designed to operate to their maximum performance without undesirable interference; and the application of duty cycles that provides some redundancy in detection of tags. Duty cycles can be also adapted to minimize power consumption.

The system is energy efficient, it can be powered with solar panels. The system has been used in remote areas with frequent cloud coverage such as in the Amazon and in places with long hours of darkness such as the winter in Tasmania (42°S).

The data formats adopted for both the system and RFID tags capture most of the high-level metadata required for an efficient data exchange. The data management within the system and with data base schema facilitates data storage.

Several examples of data sets obtained in the field were presented and discussed to demonstrate the quality of the implemented system.

While the cost of the system is important, it is equally relevant to provide the user with hardware that is easy to operate, easy to assess the health conditions of the electronics, and easy to assess the quality of the data while providing ways the data can be accessed and curated. While entomologists and beekeepers have deep expertise with hive management and bees, they are still developing their capability with electronics and ICT. Therefore, user experience is important to ensure adoption of the technology.

## Figures and Tables

**Table 1:** The encoding for CSV file names.

Field	# Characters	Content
YYYY	4	Year
MM	2	Month
DD	2	Day
CC	2 *	Country
SSS	3 *	Site
TT	2 *	Platform Type
NNN	3 *	Platform Number

* Hexadecimal value.

**Table 2:** Examples of file names and the interpretation of their meaning.

File Name	Date	Country	Site	Platform Type	Platform Number
20171022_0A00101001.csv	22 October 2017	Argentina	Tucumán	Hive	1st Hive
20170317_0D00B02001.csv	17 March 2017	Australia	Sandy Bay	Feeder	1st Feeder
20150519_1D00201006.csv	19 May 2015	Brazil	EMBRAPA	Hive	6th Hive

**Table 3:** The encoding for RFID tags. All digits are in hexadecimal, except ’Bee Number’ which is assigned to four decimal digits.

Field	Digits	Content
CC	2	Country
SSS	3	Site
TT	2	Platform Type
NNN	3	Platform Number
XXX	3	Bee Species
YY	2	Bee Strain
Z	1	Bee Type
****	4	Reserved
BBBB	4	Bee Number

**Table 4:** The table shows two examples of identification codes and their interpretation.

Parameter		Example 1		Example 2
RFID Tag Code		0D0110200100103300000005		1D0020100600202300000019
Country	0D	Australia	1D	Brazil
Site	011	Sandy Bay	002	EMBRAPA
Platform Type	02	Feeder	01	Hive
Platform Number	001		006	
Bee Species	001	*Apis mellifera*	002	*Melipona fasciculata*
Bee Strains	03	Golden Italian	02	No Strain
Bee Type	3	worker	3	worker
Reserved Digits	0000		0000	
Bee Number	0005		0019	

**Table 5:** The first three lines of sample content in the first file show the detection of three different bees (#1130, #5, #3) on the 17 March 2017 at 1:41:07 (UTC). The second file shows two different bees (#15, #19) detected on the 19 May 2015 between 9:04:17 (UTC) and 9:33:01 a.m. (UTC).

**File:** 20170317_0D00B02001.csv		
**Time in UTC**	**Bee Tag Data**	**Antenna ID** *
20170317T014107Z,	0D0080100100103300001130,	2
20170317T014107Z,	0D0110200100103300000005,	4
20170317T014107Z,	0D0110200100103300000003,	4
20170317T014108Z,	0D0080100100103300001130,	2
20170317T014108Z,	0D0110200100103300000003,	4
⋯		
**File:**20150519_1D00201006.csv **		
**Time in UTC**	**Bee Tag Data**	
20150519T090417Z,	1D0020100600202300000019
20150519T090455Z,	1D0020100100202300000015
20150519T090457Z,	1D0020100100202300000015
20150519T092035Z,	1D0020100600202300000019
20150519T093301Z,	1D0020100100202300000015
⋯		

* Details on antenna configuration are given in Section 3.3.2 and Section 3.3.3, and Figure 7. The “Antenna ID” field is optional; ** This experiment involving stingless bees in Brazil used a single antenna in the entrance housing, because direction of movement of bee activity was not required.

**Table 6:** Data sample illustrating the activity of a single drone from Argentina in a day, including an interpretation of events associated to this bee’s activity. The data interpretation is subject to the user experience, which should also consider the experiment protocol adopted. Last data of the previous day and the first data of the next day are also shown.

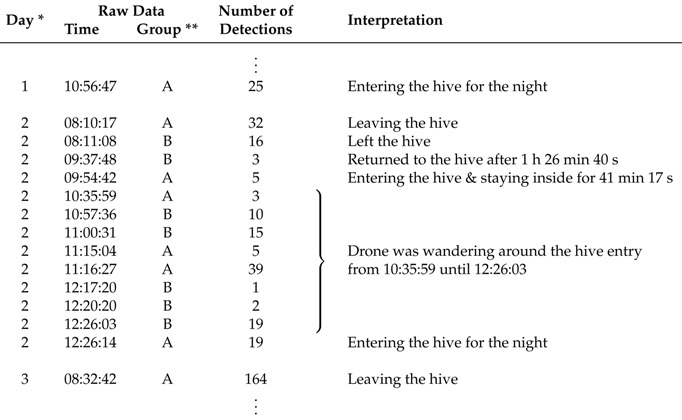

* First day is 23 October 2017; ** Group A: antennas 1 and 2 (inside; facing the hive); Group B: antennas 3 and 4 (outside; facing the environment).
